# The protective effect of vitamin D on ovarian reserve and anti‐mullerian hormone in patients undergoing chemotherapy for breast cancer, a randomized phase ΙΙ clinical trial

**DOI:** 10.1002/cnr2.2104

**Published:** 2024-06-24

**Authors:** Zahra Dastmardi, Marzieh Lashkari, Arefeh Saeedian, Mahdi Aghili, Sadaf Alipour

**Affiliations:** ^1^ Radiation Oncology Research Center, Cancer Research Institute Iran Cancer institute Tehran University of Medical Sciences Tehran Iran; ^2^ Department of Radiation Oncology Tehran University of Medical Sciences Tehran Iran; ^3^ Breast Disease Research Centre (BDRC), Arash Women's Hospital Tehran University of Medical Sciences Tehran Iran

**Keywords:** anti‐mullerian hormone, breast cancer, ovarian reserve, vitamin D

## Abstract

**Background:**

Reduced ovarian reserve is among the crucial long‐term side effects of using chemotherapy agents in breast cancer, yielding early ovarian failure. On the other hand, vitamin D is an essential factor in protecting the follicles and an important predictive factor for successful IVF therapy.

**Aim:**

The aim of this study is evaluation of vitamin D as a agent that can reduce fertility complications of chemotherapy specially in young women.

**Methods:**

Breast cancer patients undergoing chemotherapy at two cancer institutes were enrolled in this study. The case group received 1000 IU of calcitriol, and the AMH level was measured at the baseline, after chemotherapy, and six months after chemotherapy. The primary end point was improvement in the AMH level after six months of chemotherapy. the secondary endpoint was to evaluate the predictive factors of AMH level decline during chemotherapy.

**Results:**

Between 2018 and 2019, 18 and 15 patients were enrolled in the case and control groups, respectively. The mean AMH level (ngr/ml) of the patients in the case and control group were 3.16 and 2.37 ng/mL, respectively (*p*‐value = .16). These levels were 0.387 and 0.19 after six months (*p*‐value = .38). The AMH rise immediately after chemotherapy cycles to six months after chemotherapy, in the case and control groups were 0.86 and 0.44 ng/mL, respectively, which was slightly higher in the case group but not statistically significant between two groups (*p*‐value = .054).

**Conclusion:**

Despite a minimal rise in the AMH level after six months of chemotherapy, the study could not demonstrate any protective effect of vitamin D on patients' ovarian reserve undergoing chemotherapy for breast cancer. Further larger studies are needed to evaluate the effect of vitamin D supplements on ovarian reserve beside optimal dose and duration.

## 1 INTRODUCTION

Breast cancer is the most common cancer in women of their gestational age and the second cause of cancer related death in women.^1^, ^2^ As the screening programs and early detections improve the patient's survival, the micro‐metastasis is becoming a new concern. Recent trials have shown the benefits of adjuvant and neoadjuvant chemotherapy to reduce the micro‐metastasis, and therefore the relapse rate.^3^

However, various adverse side effects are reported for the use of chemotherapy agents. In addition to hair loss, nausea and vomiting, fatigue, bone marrow suppression, cognitive dysfunction, and reduced overall quality of life, changes in sexual function and fertility are among considerable common side‐effects.^4^

About 6% of breast cancers are detected in women younger than 40 years old. As the survival rate and the patients' lifespan are increasing with newer pharmacotherapeutic agents, it is essential to consider these agents' complications and effects on their quality of life, besides their effectiveness on the tumor.^5^

Despite the long‐term adverse effects, chemotherapy can reduce the growing follicles in women and decrease the ovarian reserve (OR), which results to early ovarian failure and reduced gestational period.^5^, ^6^ Protecting the OR is growing a concern in the treatment of breast cancer.

The anti‐Mullerian hormone (AMH) is glycoprotein of the TGF‐β family, showing small follicle activity. This hormone is an indirect indicator of the OR, and chemotherapy‐related follicular damage. As a parameter that is unaffected by the menstrual cycles, AMH is a reliable marker to identify the OR.^5^, ^7^ Studies suggest that high AMH level is also protective against of endometrial cancer.^8^ Endometrial cancer is often treated with total hysterectomy and bilateral salpingo‐oophorectomy (TH‐BSO) followed by adjuvant radiation therapy.^9^

Vitamin D deficiency is one of the factors associated with development of breast cancer.^10^ Vitamin D decreases tumor growth and increases CD8+ T cells infiltration in the tumor microenvironment.^11^ Recent studies have shown that vitamin D is also a key in OR level, and its deficiency has a significant impact on the failure of in‐vitro fertilization (IVF) or polycystic ovarian syndrome control. Vitamin D is among the AMH gene promoters, which can probably affect the OR, directly. Vitamin D helps producing steroids and maturation of ovarian follicles. Vitamin D regulates the number of granulosa cells and AMH signaling in cultures of ovarian follicles.^12^ One study Demonstrated a significant rise in the AMH level by a single dose of vitamin D administration, and concluded that vitamin D improved AMH production in perifollicular cycle.^13^

This study is the first study evaluates the protective effect of vitamin D on the OR of breast cancer patients undergoing chemotherapy by comparing AMH levels before and six months after chemotherapy with vitamin D supplements.

## 2 METHODS

Women aged 18–45 years old, undergoing adjuvant or neoadjuvant chemotherapy due to breast cancer from 2018 to 2019 at Imam Khomeini Cancer Institute were included as the intervention group to this study. The control group comprised women aged 18–45 years old underwent adjuvant or neoadjuvant chemotherapy for breast cancer during the same period in another cancer institute at Arash Hospital. The primary end point was the recovery of the AMH level after six months of chemotherapy. the secondary endpoint was to evaluate the factors that predict a decline in AMH level during chemotherapy.

The exclusion criteria were as follows:

- Receiving any prior cytotoxic drug.
- Having a history of any fallopian tube or ovarian surgery (such as bilateral salpingo‐oophorectomy).

Randomization was based on a web‐based computer program^14^ to generate a list with a permuted block method. After showing the group of randomizations of each participant, they enrolled from two selected hospitals.

Sample size was calculated according to a study showed an AMH level of 1.3 ng/mL post‐chemotherapy. The increase in AMH value has differed across studies. Some investigations reported the persistent low values.^15^ Henry et al. reported a recovery of 1.2% per month in AMH level.^16^ Due to vitamin D supplementation, we expected an AMH level of 1.31 ng/mL. The power of 80% and type Ι error (*α*) was .05. taking into account of 5% loss, the required sample size in each group was 20 patients.

Information regarding demographics, marital status, history of oral contraceptive use (OCP), number of pregnancies as well as tumor characteristics was collected.

The baseline AMH level was measured in all of the patients before the first chemotherapy session. Patients in the intervention group were treated with 1000 IU of calcitriol every day, from the first day of receiving chemotherapy until six months after the last cycle of chemotherapy. Their AMH level was checked on the final day of receiving chemotherapy, as well as six months after. AMH levels were measured with same kits in both groups. The satisfactory level with ELISA method was at least 2.2. The levels less than 0.7 were reported as very low fertility.

The prescribed chemotherapy regimens were following the standard protocol, based on the specialist's opinion. All of the patients were aware of the research, its method, and goals and signed the written informed consent. The study's methodology was approved in the ethics committee of Tehran University of Medical Sciences (IR.TUMS.IKHC.REC.1397.237) and registered in the Iranian Registry of Clinical Trials on March 12, 2019.

Before treatment, after treatment, and six months later, the AMH level of the patients was registered, as well as their demographic and cancer‐related data, and analyzed using IBM® SPSS 25.0.

## 3 RESULTS

Overall, 18 and 15 patients were included in the case and control groups, respectively. Patient accrual was stopped due to COVID‐19 pandemic. Their demographic data is compared in Table 1. The mean age of the patients was 34.48 (±4.12) and 35.18 (±3.81) in the case and control groups, respectively. The mean BMI of the case and control group patients was also 24.74 (±2.66) and 25.51 (±4.06), respectively. Patients in the case and control groups averaged 1.62 and 1.24 pregnancies, respectively. There was no difference in the age, marriage status, number of pregnancies, use of OCP, menstruation disturbance, or any other demographic baseline information, as well as the TN staging or IHC profile of the tumors (Tables 1 and 2).

**TABLE 1:** The demographic information of the case and control group patients.

Parameter	Cases	Controls	*p*‐value
Age	35.18 (±3.81)	34.48 (±4.11)	.590^a^
Para	1.41 (±0.87)	1.71 (±0.96)	.315^a^
BMI	24.74 (±2.66)	25.52 (±4.06)	.489^a^
Initial AMH level	3.17 (±3.31)	2.37 (±2.22)	.400^a^
Married	16 (94.1%)	18 (85.7%)	.613^b^
Use of OCP	3 (17.6%)	6 (28.6%)	.243^b^
Smoking	0 (0%)	0 (0%)	‐
Infertility	1 (5.9%)	3 (14.3%)	.609^b^
Irregular menstruation	3 (17.6%)	4 (19%)	1.00^b^
GNRH after chemotherapy	23.3%	6.7%	.33
Amenorrhea after chemotherapy	55.8%	60%	1.00

^a^ Independent t‐test.

^b^ Fisher's exact test.

**TABLE 2:** The IHC profile of the tumors of the patients in the case and control groups.

Receptor	Positive in cases	Positive in controls	*p*‐value
Estrogen (ER)	12 (70.6%)	10 (47.6%)	.197^a^
Progesterone (PR)	9 (52.9%)	9 (42.9%)	.745^a^
HER2	5 (29.4%)	7 (33.3%)	1.00^a^

^a^ Fisher's exact test.

35.3% and 64.7% of the cases underwent modified radical mastectomy (MRM) and breast conservative surgery (BCS), similar to the control group (26.7% and 73.6%; *p*‐value = .71 in Fisher's exact test). In addition, there was no difference in the chemotherapy regimen of the case and control patients, as shown in Table 3 (*p*‐value = .35 in Chi‐Square test).

**TABLE 3:** The chemotherapy regimen of the patients in case and control groups.

Regimen	Cases	Controls	*p*‐value
AC‐T	16 (88.2%)	12 (81.0%)	.35^a^
AC4	1 (5.9%)	1 (4.8%)	
TC	1 (5.9%)	2 (14.3%)	

^a^ Chi‐square test.

17.6% of the cases experienced at least one chemotherapy‐related adverse effect, which was statistically similar to the 26.7% rate in the control group (*p*‐value = .67 in Fisher's exact test). Neutropenia was the most common adverse effect (data not shown).

In the follow‐up, 55.8% of the patients receiving vitamin D and 60% of the patients in the control group, had amenorrhea six months after the chemotherapy (*p*‐value = 1.00 in Fisher's exact test). Similarly, AMH levels were not statically significant between the case and control arm, before, after, and six months after chemotherapy (Table 4). However, the increase in AMH level after six months of chemotherapy cycles was higher in the case group compared to the level immediately after chemotherapy. This difference, however, was not statistically significant when compared to the control group (0.87 and 0.44 respectively; *p*‐value = .054 in independent t‐test).

**TABLE 4:** The AMH level (ngr/ml) of the patients before, immediately after, and 6 months after chemotherapy in case and control groups.

AMH Level	Cases	Controls	*p*‐value
Before Chemotherapy	3.16	2.37	.16^a^
Immediately After Chemotherapy	0.082	0.35	.54^a^
6 Months After Chemotherapy	0.387	0.19	.38^a^
Decline during chemotherapy	3.00	1.38	.11^a^
Rise after chemotherapy	0.87	0.44	.054^a^

^a^ Independent t‐test is performed.

The subgroup analysis showed a significant correlation between age and the decline in AMH levels (Pearson's r = −0.46, *p*‐value = .008). The AMH level would decrease significantly with the age, which can be reported as a significant decline in the OR in older patients. Additionally, the reduction in AMH level during chemotherapy was significantly greater in patients with higher levels before chemotherapy (*p*‐value = .00, Pearson: 0.985). There was no correlation between mean reduction in AMH level and taking vitamin D regarding chemotherapy regimen before and after chemotherapy (*p*‐value = .88 in independent t‐test), BMI (*p*‐value = .62), experiencing amenorrhea (*p*‐value = .65), and stage (*p*‐value = .91), before and after chemotherapy.

## 4 DISCUSSION

Few studies have evaluated the pharmacological ovarian protection during chemotherapy. This longitudinal case–control study was the first to evaluate the protective effect of vitamin D, in 6 months for the first time. In terms of this study's power, no statistically significant difference was detected in the patients' AMH level before their first chemotherapy course and immediately after chemotherapy, and six months later than the last course in patients receiving vitamin D supplementation and the control group.

Breast cancer is suggested to affect follicles and reduce the OR, even before chemotherapy initiation.^17^ However, older age, as well as higher BMI and smoking, are shown as risk factors, making patients' OR more susceptible to chemotherapy.^15^ In this study, the case and control group had similar BMIs (*p*‐value = .489) and none of the patients were smoker.

The study of Silva and colleagues^18^ have shown that the GnRH addition to the chemotherapy regimen can be an accelerating factor for post chemotherapy ovarian recovery. However, more reliable indicators, such as the AMH level, are needed to be monitored.

The short follow‐up period can be a reason for the inconclusive results of this study. The Anne Perdrix study^15^ on the use of Taxan have shown that AMH level kept increasing until three years. However, this study only showed an improvement in mean AMH rise (0.87 and −0.044 respectively; *p*‐value = .054 in independent t‐test), which was not statistically significant and there was no significant difference in the AMH increase of patients with or without Taxan.

Vitamin D deficiency is common in young women, and it is necessary to repeat the study with a larger population size, as the protective effect of vitamin D is proved in advance.^12^ Also, longer follow‐up and checking other OR markers are needed to reach conclusive outcomes.

Our study has several limitations that must be considered. The small sample size of each arm has decreased the study's power to detect significant differences. To the best of our knowledge, this is the first study to evaluate the effect of vitamin D supplements on recovering AMH levels in patients receiving chemotherapy. Nonetheless, larger populations are needed to make more reliable approvements or rejections in this concern.

The study could not check the baseline vitamin D level of the patients, due to ethical issues. Low vitamin D levels in the control arm must be treated ethically. However, extended methodologies can improve this point to reduce the potential confounders.

Different doses can also be checked, besides evaluating the metabolites of vitamin D to completely study its effects.

## 5 CONCLUSION

As the first study, investigating the protective effect of vitamin D on ovarian reserve of the breast cancer patients, there was a slightly higher mean AMH rise in patients receiving 1000 IU calcitriol. However, we found no significant differences in the immediate and after six months AMH level of patients undergoing chemotherapy, with or without vitamin D therapy.

This pilot study warrants further investigation with larger population sizes and longer follow‐ups to reach conclusive outcomes.

## AUTHOR CONTRIBUTIONS

**Zahra Dastmardi:** Formal analysis (equal); investigation (equal); methodology (equal); project administration (equal); visualization (equal); writing – review and editing (equal). **Marzieh Lashkari:** Conceptualization (equal); investigation (equal); methodology (equal); project administration (equal); resources (equal); supervision (equal); writing – review and editing (supporting). **Arefeh Saeedian:** Formal analysis (equal); investigation (equal); methodology (equal); writing – original draft (lead); writing – review and editing (lead). **Mahdi Aghili:** Conceptualization (equal); data curation (equal); investigation (equal); methodology (equal); supervision (equal); writing – review and editing (supporting). **Sadaf Alipour:** Investigation (equal); methodology (equal).

## CONFLICT OF INTEREST STATEMENT

The authors whose names are listed on the title page and shared in the manuscript, certified that they have no affiliations with or involvement in any organization or entity with any financial or non‐financial interests.

## INFORMED CONSENT STATEMENT

Informed consent was obtained from all subjects involved in the study.

## ETHICS STATEMENT

The current study maintained a standard of ethical conduct and was overseen by the Ethical Committee of Tehran University of Medical Science, with approval number IR.TUMS.IKHC.REC.1397.237.

## Data Availability

The datasets used and analyzed during the current study are available from the corresponding author upon reasonable request.
